# From data silos to actionable insights: a comparative evaluation of data access models and practical guidelines for animal health surveillance

**DOI:** 10.3389/fvets.2026.1962800

**Published:** 2026-09-17

**Authors:** Saba Noor, Ruben Taelman, Jeroen Degroote, Bart Pardon, Gerdien van Schaik, Muhammad Rafiq, Camille Delavenne, Enhong Liu, Hanlu Zhang, Puchun Niu, Miel Hostens

**Affiliations:** 1 Department of Animal Sciences and Aquatic Ecology, Faculty of Bioscience Engineering, Ghent University, Ghent, Belgium; 2 Department of Internal Medicine, Reproduction, and Population Medicine, Faculty of Veterinary Medicine, Ghent University, Ghent, Belgium; 3 IDLab, Department of Electronics and Information Systems, Ghent University-IMEC, Ghent, Belgium; 4 Department of Population Health Sciences, Unit Farm Animal Health, Utrecht University, Utrecht, Netherlands; 5 Faculty of Engineering Technology, KU Leuven, Brugge, Belgium; 6 EpiMundi, Lyon, France; 7 Department of Animal Science, Cornell University, Ithaca, NY, United States

**Keywords:** animal health surveillance, data sovereignty, fair principles, federated data access, ontologies, solid pods, veterinary epidemiology

## Abstract

**Background:**

Effective livestock disease surveillance depends on scalable, privacy-conscious access to distributed diagnostic data, yet current systems are constrained by fragmented datasets, inconsistent formats, and limited interoperability.

**Objective:**

This paper compares three approaches for accessing distributed animal health data: direct data sharing, centralized access, and federated access using Solid Pods. The comparison covers architectural properties, query performance benchmarks, and the FAIR (Findable, Accessible, Interoperable, Reusable) maturity of the data artifacts produced by each approach, with the aim of providing practical guidance for choosing the right strategy in livestock health surveillance.

**Methods:**

The three approaches were characterized along seven operational criteria: data access mechanism, privacy and governance, integration workflow, consistency, workflow scalability, ease of use, and total cost of ownership, using the European Cattle Barometer as a testbed. Two experiments measured query performance: centralized versus federated SPARQL on 76,295 cattle records (740,056 RDF triples) across five Solid Pods, and vertical versus horizontal federation across 12 Pods. Queries were mapped to the Livestock Health Ontology (LHO), run five times, with differences tested using Welch’s t-test. FAIR maturity was scored against the 41 indicators of the Research Data Alliance (RDA) FAIR Data Maturity Model.

**Results:**

Across the seven operational criteria, direct sharing scored high only on ease of use but low on scalability, privacy, integration, and consistency. Centralized access scored highest on data consistency and ease of automation, while federated access scored highest on workflow scalability, privacy and governance, and data integration. Centralized queries finished in 20.2 ± 4.1 s versus 30.0 ± 2.2 s for federated, 1.49 times slower (*p* = 0.003). Vertical and horizontal federation performed comparably (*p* = 0.060) and returned identical results (1,378 records). FAIR maturity of the published data was 6%, 60%, and 83% for direct, centralized, and federated access, respectively.

**Conclusion:**

No single approach suits all situations: direct sharing for short-term simplicity, centralized access for fast dashboards, and federated access where data ownership and FAIR-aligned outputs matter. In particular, the choice between centralized and federated access is a trade-off between query speed and data sovereignty. These findings inform practical, scalable, and privacy-conscious data-sharing guidelines for livestock disease surveillance.

## 1 Introduction

In animal health surveillance, the value of data-driven decision-making depends not only on the availability of large datasets but also on the mechanisms enabling their integration, sharing, and reuse. In One Health surveillance research, the persistence of “data silos” and the lack of interoperability remain central obstacles, slowing analytical workflows and undermining the scalability of digital infrastructures (1, 2). Recent studies of integration architectures in smart agri-food information systems also identify interoperability, semantic alignment, and governance constraints across heterogeneous data sources as the dominant obstacles to cross-organizational data reuse (3). Efforts to operationalize FAIR (Findable, Accessible, Interoperable, Reusable) principles in animal farming data management, for example, across dairy and fish-farming projects, have shown that secure access protocols, semantic interoperability, and metadata standards are non-trivial to deploy even within a single coordinated project (4). At the policy level, the emergence of EU Common European Data Spaces for agri-food explicitly contrasts top-down centralized standardization against decentralized, federated data-exchange architectures, and frames data-access architecture as a key determinant of whether FAIR properties survive across data-sharing chains (5). These challenges are acute in veterinary epidemiology, where relevant data are distributed across a wide range of sources, including diagnostic laboratories, veterinary practices, governmental and industry databases, slaughterhouses, and farms, and are often stored in heterogeneous formats and governed by diverse legal and institutional policies (6, 7). As in other privacy-sensitive domains, issues of confidentiality, ownership, and stakeholder trust further restrict the sharing of sensitive datasets (8).

Centralized data access approaches, such as those implemented in national surveillance programs in Netherlands (9, 10) and France (11), and in the centralized phase of the Cattle Barometer, a cross-country dashboard integrating laboratory-confirmed bovine respiratory disease diagnostic data from multiple European laboratories (12, 13)show that aggregating data into a single repository can enhance integration and support sophisticated analytical dashboards. However, these systems are hard to scale: they depend on a single central server as a point of failure, need case-by-case re-engineering to onboard each new data provider (6, 14), and raise General Data Protection Regulation (GDPR) and data-ownership concerns that labs are often reluctant to accept (8, 15). These limits motivate the federated alternatives evaluated in this study, which preserve data providers’ autonomy by enforcing access control at each data source rather than at a central custodian (16).

Federated access, particularly approaches based on Personal Online Data Stores (Pods) developed by Tim Berners-Lee (16, 17), offers an alternative by enabling in-place querying of distributed datasets while preserving data sovereignty. Comparable federated infrastructures have been demonstrated for distributed analytics in human healthcare (18) and for life sciences data sharing more broadly (19, 20). Despite their conceptual promise and adoption in other fields (18–20), no study has yet empirically compared direct, centralized, and federated access on the same real animal-health data, and evidence on the technical performance of federated querying relative to centralized systems remains particularly limited. Within livestock specifically, recent work has proposed decentralized trust frameworks for secure supply-chain data exchange (21) and federated learning, distributed model training, not data querying, for cattle health monitoring from IoT sensor data (22); neither addresses federated data access via query languages such as SPARQL, benchmarks query-response performance across access architectures, or assesses FAIR data maturity, the combination this study provides. This study offers a comparative technical evaluation of three data access approaches for animal health surveillance: (i) direct data sharing, in which laboratories manually exchange diagnostic files (CSV/Excel) with the data analyst, and the analyst performs cleaning, harmonization, and integration on each file individually; (ii) centralized access, in which laboratory data are automatically ingested into a single central repository where harmonization, anonymization, and ontology mapping are performed once via a managed pipeline; and (iii) federated access, in which SPARQL (a standard query language for linked data) queries are executed in parallel over lab-controlled Solid Pods (secure, access-controlled data vaults that each laboratory hosts and manages independently). In both centralized and federated architectures, data were mapped to the Livestock Health Ontology (LHO) to support semantic interoperability. LHO is a shared vocabulary that gives all laboratories a common data structure. The three approaches are compared against seven operational criteria (data access mechanism, privacy and governance, consistency, integration workflow, workflow scalability, ease of use and implementation, and total cost of ownership), complemented by empirical query-performance benchmark tests. As FAIR (Findable, Accessible, Interoperable, Reusable) principles describe properties of data and digital objects (23, 24), rather than properties of access architectures, the FAIR maturity of the data artifact produced by each approach (the concrete data output it generates, that is, the dataset together with its accompanying metadata) is assessed separately against the 41 indicators of the Research Data Alliance (RDA) FAIR Data Maturity Model (25). This standardized framework enables a quantitative comparison of how well each approach’s published data conform to community FAIR criteria. To our knowledge, this is the first empirical comparison of direct, centralized, and federated data access in animal health surveillance, evaluated on the same real multi-laboratory data across four countries and three livestock species. The findings provide a basis for practical guidance for selecting a data access strategy in animal health surveillance.

## 2 Materials and methods

### 2.1 Data sources and case studies

This study builds on the Cattle Barometer, a surveillance tool developed within the DECIDE project to collect diagnostic test results for bovine respiratory disease (BRD) across multiple European laboratories (12, 26). From 2016 to 2023, a total of 76,295 records for 7 BRD pathogens: *Bovine coronavirus*, *Bovine respiratory syncytial virus*, *Bovine parainfluenza virus type 3*, *Histophilus somni*, *Mycoplasma bovis*, *Mannheimia haemolytica*, and *Pasteurella multocida* were collected from veterinary laboratories located in Belgium, Netherlands, Ireland, and France. The raw data, originally delivered as spreadsheets (CSV/Excel), included information on sample type, diagnostic test, pathogen, result, and location (province, country). The case study was extended with additional species, pig and poultry diagnostic data, processed through the same harmonization and mapped to Livestock Health Ontology (LHO) as RDF (Resource Description Framework), a standard for representing data as linked statements. The pig data contain 23,249 PCR test results from Spain covering 2020–2023. They track key respiratory agents, mainly the *Swine influenza virus*, *Mycoplasma hyopneumoniae*, and *Lawsonia intracellularis*, and include routine fields such as sample type (e.g., tissue, fluid, swab, feces), production stage (finishing, sow, nursery, weaning) (27). The poultry data comprise 1,889 records from 2014 to 2022, including *Infectious Bronchitis (IB)* strains (e.g., *QX*, *793B*, *D274*, *D181*). To ensure comparability across access models, the datasets were cleaned, anonymized, and harmonized into a common schema (i.e., mapping each lab’s differing field names, codes, and terminology to a single shared structure), using a dedicated data-processing pipeline developed within the DECIDE project (28), which served as the baseline input for all three data access approaches.

### 2.2 Data access approaches

Figure 1 presents the classification of the three data access approaches considered in this study: direct sharing, centralized access, and federated access. For the centralized and federated approaches, the harmonized data described in section 2.1 were additionally mapped to the LHO) and serialized as RDF, a graph-based format that represents data as subject–predicate–object statements called triples, where each triple encodes one attribute of a record. Mapping templates, example shapes, and queries for the LHO/RDF conversion are available in the LHO repository (29). Each approach is described in detail in the following subsections. The technologies underlying each approach were selected based on suitability for the use case, interoperability, and compatibility with existing DECIDE project infrastructure. Because the harmonized data is mapped to a shared ontology (the Livestock Health Ontology), RDF was selected as the underlying data representation: as a graph-based, subject-predicate-object format explicitly designed to encode ontology-structured, semantically linked data, RDF and its associated tooling (e.g., SPARQL, Comunica) are well suited to representing and querying ontology-mapped diagnostic records across heterogeneous laboratory sources. Comunica was selected as the federated query engine because it is open-source, actively maintained, and modular. Solid Pods were chosen because they enable each laboratory to retain its data in its own distributed storage rather than a central repository, are built on an open, freely accessible platform, natively support RDF and linked-data storage consistent with the ontology-mapped representation already adopted, and implement W3C standards for identity and access control (WebID, ACL). R or Python script, and Quarto supported the shared data-cleaning, anonymization, and ontology-mapping pipeline underlying both approaches: R or Python implements the cleaning and mapping logic, while Quarto automates execution whenever new laboratory files arrive, avoiding manual reprocessing. Any general-purpose business-intelligence tool could be used for visualization; Tableau was chosen here because it was already freely accessible to the team through an existing institutional license. Azure Functions were used for the federated query API to match the DECIDE project’s existing cloud infrastructure and provide serverless scalability for the query engine itself without dedicated server provisioning. Direct.

**Figure 1:**
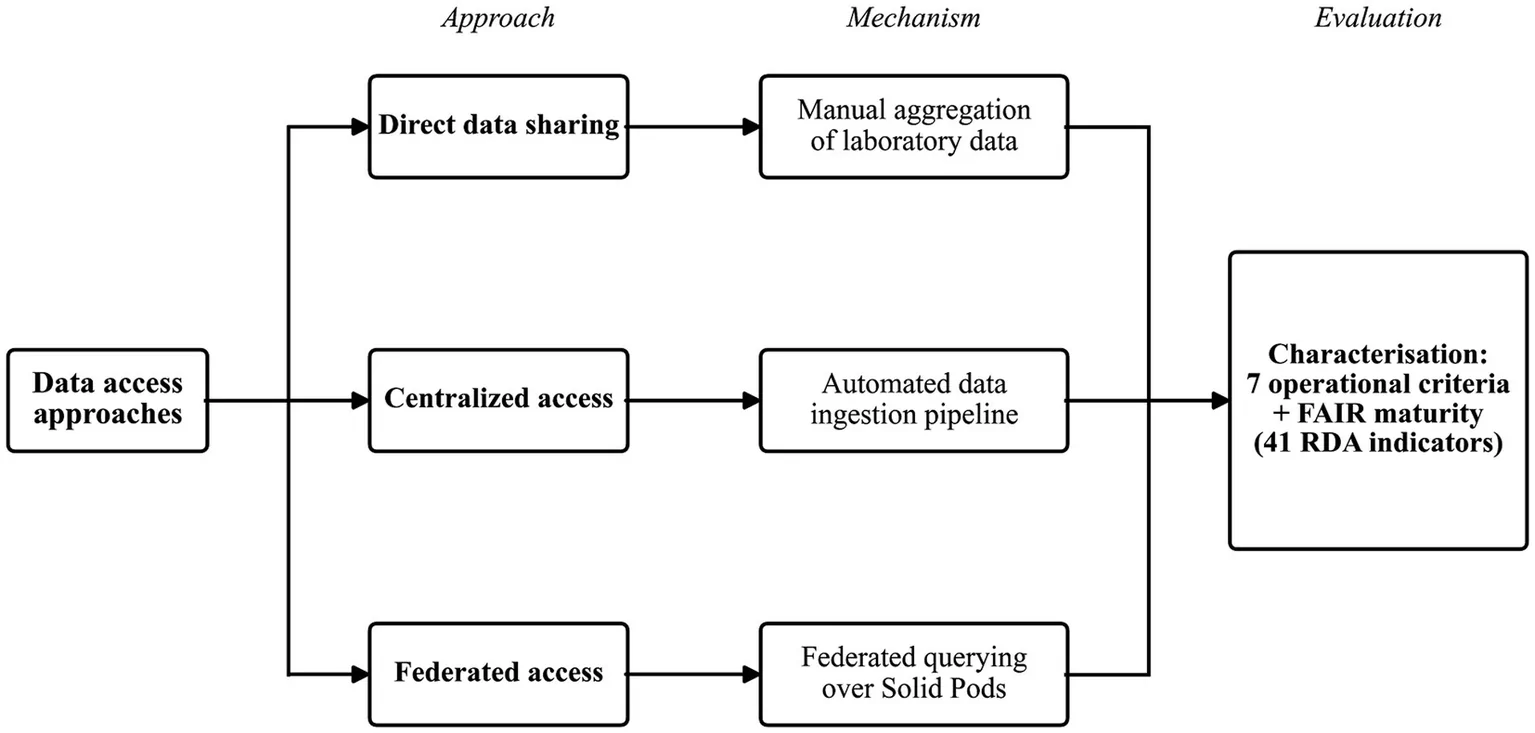
Classification of the three data access approaches, their data-handling mechanism, and their evaluation.

#### 2.2.1 Direct data sharing approach

Direct data sharing refers to the manual or semi-manual exchange of data between data providers and data analysts, typically via spreadsheets or CSV files sent by email or uploaded to shared drives. This is often the approach used by default to share data in research projects and was the initial foundation of the Cattle Barometer use case (12). The initial tutorial developed for the first version of the tool can be accessed at https://decide-project-eu.github.io/case-studies-website/tutorials/cattle-barometer.html. As shown in Figure 2, the workflow involved cleaning raw data using widely adopted tools such as statistical programming environments (e.g., R) or general-purpose scripting languages (e.g., Python), with results stored in static formats that lacked structured access control. Aggregated insights were then visualized using a commercial business-intelligence dashboard tool (e.g., Tableau). As indicated by the return (backward) arrow in Figure 2, this cycle must be repeated manually each time a new batch of data is received, since every update requires the data to be cleaned, harmonized, and integrated again from the beginning (30).

**Figure 2:**
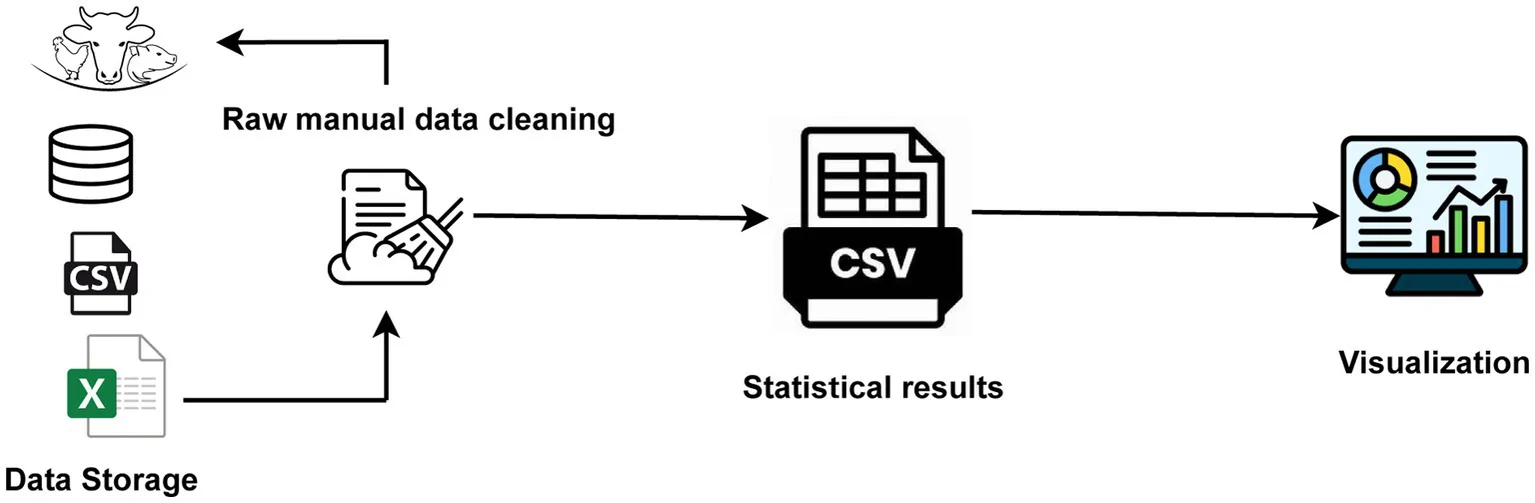
Direct data access workflow, showing manual aggregation and cleaning of laboratory data into CSV files before visualization, adapted from (30). License: CC BY 4.0. Source: Burleigh Dodds Science Publishing Open Access page.

#### 2.2.2 Centralized access

In centralized data access, the workflow is transitioned from manual exchange to a unified, automated infrastructure. Laboratories submit anonymized diagnostic results under a data-transfer agreement, formatted per a shared data script, as CSV/Excel files retrieved through a dedicated API (28); once received, this automatically triggers the R/Quarto pipeline that performs cleaning, harmonization, and mapping without further manual intervention, reducing human error and improving scalability. Access to the Cattle Barometer’s centralized repository and dashboard, the case study evaluated in this study, is administered by Ghent University, under the DECIDE project’s data management lead, who grants and revokes read and download permissions on a per-collaborator basis; access and changes to the underlying data and code are logged through the repository’s version-controlled infrastructure.

As illustrated in Figure 3, the standardized datasets were integrated into a central computing environment for downstream analysis. The raw laboratory data, typically in CSV, PDF, or Excel format, were processed using custom R scripts that performed filtering, renaming, mapping, and variable normalization. The outputs were merged into a single flat-file CSV with ten standardized fields, enabling compatibility with visualization platforms such as Tableau. To ensure semantic interoperability, the integrated dataset was also converted to RDF and mapped to the Livestock Health Ontology (LHO) (31). Query outputs from the RDF store could then be accessed programmatically or visualized interactively in Tableau. Source code for this workflow is available at https://github.com/decide-project-eu/cattle-use-case-barometer.

**Figure 3:**
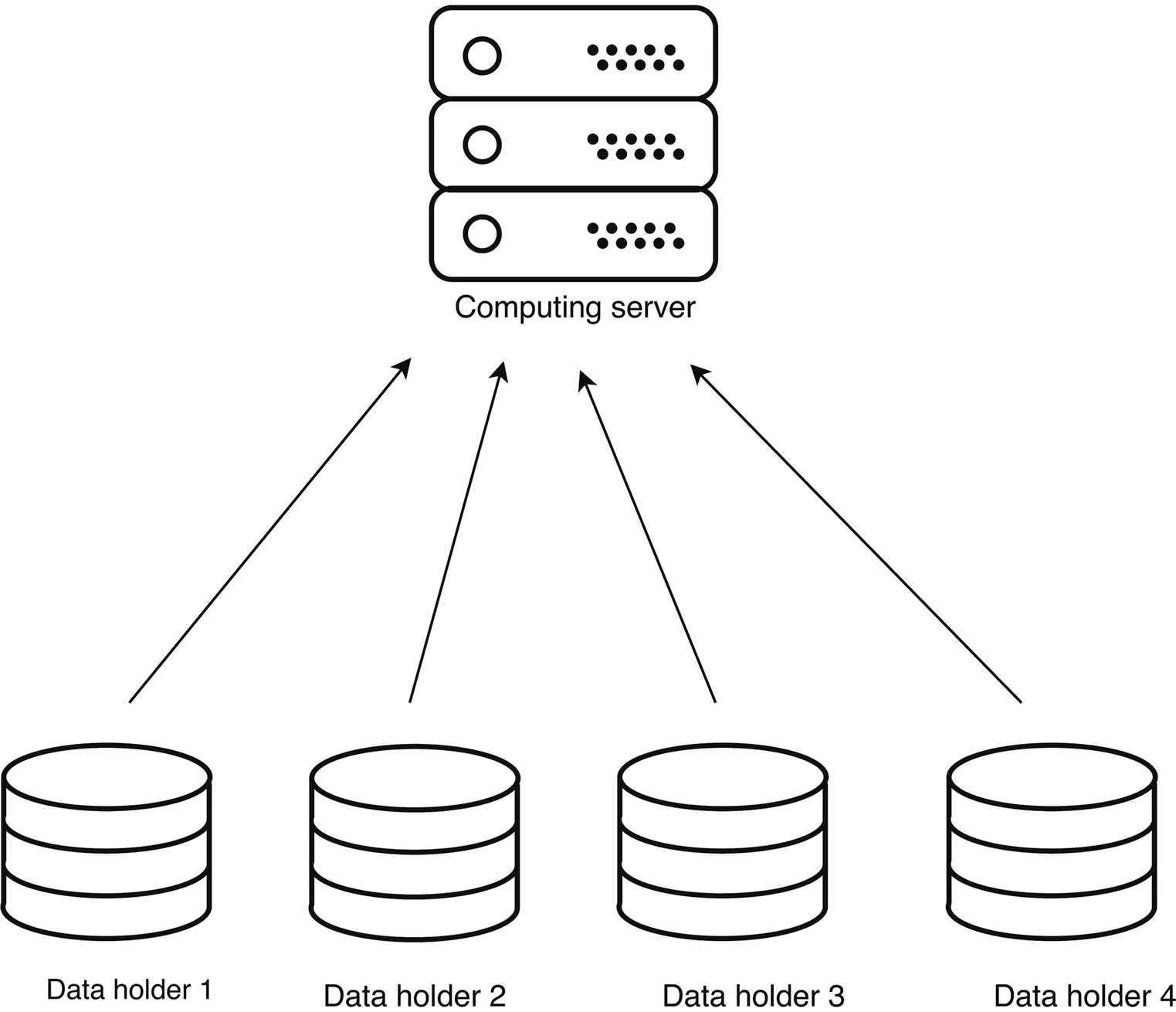
Centralized data access workflow, where multiple data silos transfer data into a single computing server.

#### 2.2.3 Federated access: decentralized approach using pods

In contrast to the centralized architecture, federated access using Pods emphasizes data ownership, security, and in-place querying of distributed data (16). Instead of aggregating all diagnostic data into a single repository, this model enables each laboratory to retain control over its own data. Each lab’s data is stored on Solid Pods, personal online data stores developed using W3C web standards for identity, access control, and interoperability (17) as shown in Table 1. Pods are decentralized web storage that offer a standards-based mechanism for data publishing and sharing, empowering data providers to manage their information while enabling structured collaboration. Access grants are issued and revoked directly by each lab owner via their Pod’s WebID-based ACL (Table 1), which specifies read, append, write, and control permissions per requester; because grants are tied to an individual WebID rather than a shared credential, every access event is inherently traceable to the requesting party, providing governance accountability by construction.

**Table 1:** Access control list (ACL) settings for demo samples (public-read) and the pod owner (data provider).

ACL rule	Applies to	Principal	Read	Append	Write	Control
.acl#public (sample data)	Resource	Everyone	✓	–	–	–
.acl#owner (provider)	Resource + default (contained)	Lab owner WebID (…/profile/card#me)	✓	✓	✓	✓

“✓” means granted; “—” means not granted. “Resource + default” means acl:accessTo to the container and acl:default for items inside it. Public read applies only to demo/sample TTLS files; production Pods remain restricted.

This federated access was implemented using Bovi-Analytics Pods^1^. Federated queries were executed using the open-source Comunica query engine^2^, accessed through a dedicated API endpoint^3^, as shown in Figure 4. Comunica query engine dynamically queries the RDF datasets hosted on the Bovi-Analytics Pods at runtime, without pre-aggregation or relocating the data (32).

**Figure 4:**
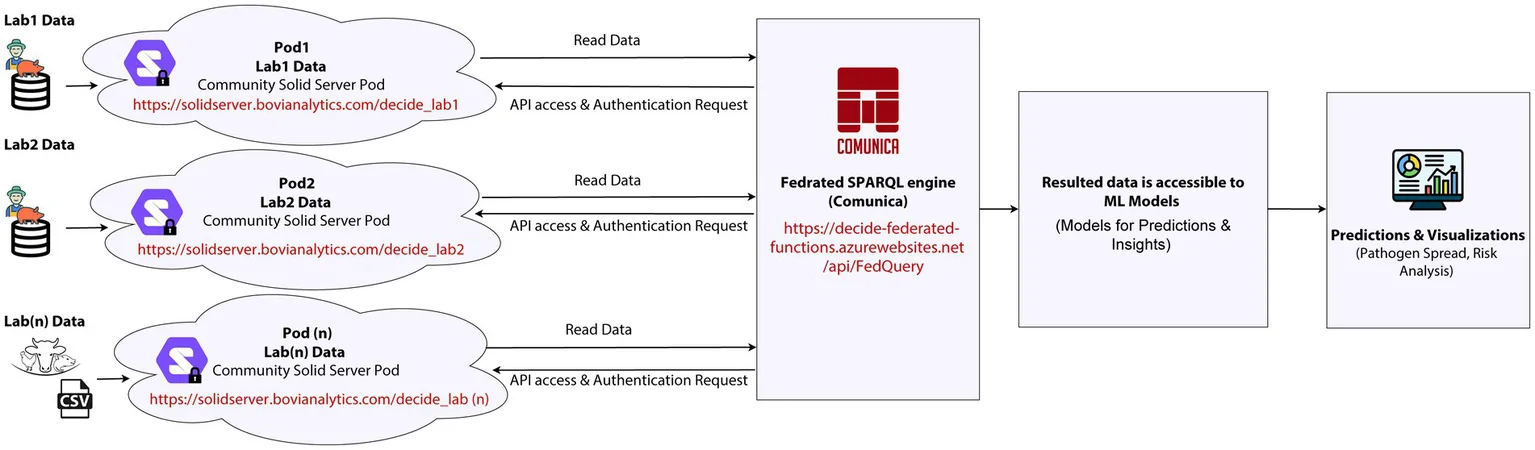
Decentralized data access workflow, using solid pods and a federated SPARQL engine to query distributed datasets for predictions and visualization.

Each pod exposes livestock diagnostic data in RDF format, a graph-based structure for encoding knowledge using subject, predicate, and object triples. For example, a single triple might record that a specific sample tested positive for a given pathogen: Sample_123, LHO:hasPathogen, LHO: MB, meaning “Sample_123 (subject) has pathogen (predicate) *Mycoplasma bovis* (object), with additional triples recording that same sample’s type, diagnostic test, country, and result. These are semantically aligned with the LHO to ensure a shared vocabulary across species and labs. When a query is submitted through the API, the engine traverses each pod, extracts the relevant RDF triples based on the defined filters, and assembles the results into a structured response that can be processed subsequently.

### 2.3 Characterization of data access approaches

The three access approaches were evaluated along the seven operational criteria defined below; the full per-approach characterization is provided in Supplementary Data Sheet S1. These seven criteria are assessed qualitatively: each approach is rated high, moderate, or low based on its design properties and our implementation experience, rather than by a numeric or weighted scoring rubric. Ratings were assigned through joint discussion and consensus among the authors, with the corresponding author finalizing the classification based on that discussion, to limit the influence of any single individual’s assessment. FAIR maturity of the data generated by each approach is assessed separately: a qualitative analysis of how each approach addresses the FAIR principles is provided in Supplementary Data Sheet S2, and a quantitative assessment against the RDA FAIR Data Maturity Model (41 indicators) in Supplementary Data Sheet S3. The seven operational criteria are:

*Data access mechanism*: The data access mechanism refers to how a data analyst obtains data. This may range from manual file exchanges between providers and analysts, through automated access via a central interface, to in-place queries against a federated query endpoint that retrieves data from multiple distributed sources (6, 16, 17).

*Data privacy and governance*: This criterion captures where access control is enforced. Privacy may be managed manually through bilateral agreements between providers and analysts, centrally by a single data custodian, or per-source via Access Control Lists (ACLs) at each provider’s data store (8, 15).

*Data integration workflow*: The integration workflow refers to where data harmonization occurs. Harmonization may be performed analyst-side (after data is received), centrally during ingestion into a managed pipeline, or in-place at query time using a shared ontology to align data semantically across sources (6, 14).

*Data consistency*: Data consistency refers to how versioning and structural alignment are maintained across providers and updates. Inconsistent versions or schemas across sources can introduce errors in downstream analyses; consistency is improved by automated pipelines, version control, or schema-enforced data publishing (14).

*Workflow scalability*: Workflow scalability refers to how operational effort grows with the number of laboratories or data sources. A scalable workflow can onboard new providers with minimal incremental effort, whereas a non-scalable workflow requires manual re-engineering for each new source (3, 6).

*Ease of use and implementation*: This criterion captures the setup cost and ongoing operational effort required for both data providers and analysts. It includes the technical expertise needed to deploy the system, the learning curve for end-users, and the maintenance burden over time.

*Total cost of ownership (TCO)*: TCO captures the personnel, infrastructure, and maintenance costs over the system’s lifecycle. It includes one-time setup costs, recurring infrastructure costs (e.g., servers, storage, query engines), and ongoing maintenance effort (e.g., software updates, ontology evolution, ACL management).

### 2.4 Experiment setup for performance and scalability test

#### 2.4.1 Experiment 1: comparison of a centralized versus federated query response time on the DECIDE cattle respiratory barometer dataset

To benchmark data access architectures, an experiment was designed to compare query response time of centralized and federated querying approaches in terms of query response time (mean and standard deviation over five runs) and result correctness (whether both architectures return the same row set), executed on the same cattle respiratory barometer dataset (76,295 records/740,056 RDF triples) with the same SPARQL query (Q1). In the centralized setup, each lab’s raw Excel files were initially processed using dedicated R scripts for cleaning, filtering, SHA-256 anonymization (a one-way cryptographic technique that converts sensitive identifiers, such as farm IDs, into irreversible codes), and mapping to a standardized cattle schema^4^. This process was automated through a GitHub-managed Quarto pipeline that detects incoming files, transforms them, and exports the cleaned data directly to the Barometer dashboard. For the production dashboard,the final dataset, consisting of 76,295 records across 10 standardized columns (~27.35 MB), was exported as a flat .csv file and integrated with Tableau^5^. For the query-time benchmark, the same cattle data (740,056 RDF triples) were consolidated into a single RDF file hosted on a Solid Pod and queried with Comunica (@comunica/query-sparql v5.2.0) using query Q1.

In the federated setup, the same cattle respiratory barometer dataset converted to RDF was uploaded to five Solid Pods hosted at https://solidserver.bovi-analytics.com/, with controlled read access managed via ACLs. Each lab maintained its RDF data independently, uploaded via the PENNY tool^6^, and structured using the Livestock Health Ontology (LHO) to ensure semantic consistency. The RDF triple counts per lab were as follows: Lab 1: 227,310 triples, Lab 2: 148,574 triples, Lab 3: 267,168 triples, Lab 4: 5,980 triples, Lab 5: 91,024 triples, and Total RDF triples across all five cattle Pods: 740,056. A federated SPARQL query (Q1) was executed targeting *Mycoplasma bovis*-positive swabs, as shown below in Table 2.

**Table 2:** Federated SPARQL query (Q1).

PREFIX LHO: <http://www.purl.org/decide/LiveStockHealthOnto/LHO#>
SELECT? Sample? Pathogen? Breed? SampleType? DiagnosticTest? Country? Province? SampleResult
WHERE
{
? Sample a LHO: CattleSample;
LHO:hasPathogen? Pathogen;
LHO:hasSampleType? SampleType;
LHO:hasCountry? Country;
LHO:hasProvince? Province;
LHO:hasBreed? Breed;
LHO:hasDiagnosticTest? DiagnosticTest;
LHO:hasResult? SampleResult.
FILTER (? Pathogen = LHO: MB)
FILTER (? SampleType = LHO: Swab)
FILTER (? SampleResult = LHO:1)
}

Executed against the five cattle Solid Pods, this query retrieves all cattle samples testing positive for *Mycoplasma bovis* (MB) from swab specimens, returning each matching sample’s pathogen, breed, sample type, diagnostic test, country, province, and result.

Queries were executed using the Comunica SPARQL engine (33) (“@comunica/query-sparql” v5.2.0) deployed as an Azure Function on an App Service Standard plan (S2: 2 dedicated cores, 3.5 GB RAM). Earlier Comunica versions exhibited query failures (HTTP 500 errors) at larger data volumes; v5.2.0 incorporates an upstream federation-planning optimization that we helped validate, and all experiments reported here use this corrected version. The engine fetches TTL files from each Solid Pod. Each query was executed five times to compute the mean and standard deviation. Differences in query time were assessed with a two-sample Welch’s t-test (unequal variances) in Python, with significance at α = 0.05 (N = 5 runs per configuration). No pre-aggregation was performed, and all data remained in their original Pods. For demonstration purposes, the complete implementation has been uploaded to the GitHub repository and is available here^7^.

#### 2.4.2 Experiment 2: comparison of federation scalability for vertical vs. horizontal data access

In this experiment, we expanded the number of Pods from five Pods in the previous experiment to twelve Pods, and included data from additional species (cattle, pigs, and poultry) to test the scalability of federated access. The aim was to observe how performance varies as the data volume and pod count grow, reflecting real-world scenarios of distributed veterinary diagnostics.

Two types of federation were tested: vertical (species-specific) and horizontal (mixed species). For vertical federation, Pods 1 to 5 represented cattle data from five separate labs, Pods 6 to 11 held pig data from six different labs, and Pod 12 contained poultry data. Although each group was species-specific, the datasets across labs were still heterogeneous, with differences in format, structure, and terminology. These variations were semantically harmonized using the LHO to enable consistent federated querying across vertically aligned Pods. In the horizontal federation setup, the same data was redistributed across six mixed-species Pods, with each pod containing combined cattle, pig, and poultry data, resulting in even greater heterogeneity in both content and ontology structure complexity. Both test types used the same complex SPARQL UNION query (Q2), targeting *Mycoplasma bovis*-positive swabs (cattle), *Mycoplasma hyopneumoniae*-positive tissue samples from finishing pigs, and *Infectious Bronchitis QX*-positive samples from broiler poultry. This query retrieves matching records from all three species in a single federated request, as shown below in Table 3.

**Table 3:** Federated SPARQL UNION query (Q2).

PREFIX LHO: <http://www.purl.org/decide/LiveStockHealthOnto/LHO#>
SELECT? Sample? Species? Pathogen? Breed? SampleType? DiagnosticTest? ProductionStages? Country? SampleResult
WHERE {
{
? Sample a LHO: CattleSample;
LHO:hasPathogen? Pathogen;
LHO:hasCountry? Country;
LHO:hasResult? SampleResult;
LHO:hasBreed? Breed;
LHO:hasSampleType? SampleType;
LHO:hasDiagnosticTest? DiagnosticTest.
FILTER(? Pathogen = LHO: MB)
FILTER(? SampleType = LHO: Swab)
FILTER(? SampleResult = LHO:1)
BIND(“Cattle” AS? Species)
}
UNION
{
? Sample a LHO: PigSample;
LHO:hasPathogen? Pathogen;
LHO:hasCountry? Country;
LHO:hasResult? SampleResult;
LHO:hasBreed? Breed;
LHO:hasSampleType? SampleType;
LHO:hasDiagnosticTest? DiagnosticTest;
LHO:hasProductionStages? PigProductionStage.
FILTER(? Pathogen = LHO: Mycoplasma_hyopneumoniae)
FILTER(? SampleType = LHO: Tissue)
FILTER(? PigProductionStage = LHO: Finishing)
FILTER(? SampleResult = LHO:1)
BIND(“Pig” AS? Species)
}
UNION
{
? Sample a LHO:PoultrySample;
LHO:hasPathogen? Pathogen;
LHO:hasBreed? Breed;
LHO:hasSampleType? SampleType;
LHO:hasProductionStages? PoultryProductionStage;
LHO:hasCountry? Country;
LHO:hasResult? SampleResult
FILTER(? Pathogen = LHO: QX)
FILTER(? SampleType = LHO: SS)
FILTER(? PoultryProductionStage = LHO: Broiler)
FILTER(? SampleResult = LHO:1)
BIND(“Poultry” AS? Species)
}
}

Executed across the twelve (vertical) or six (horizontal) Pods, this query retrieves pathogen-positive samples across three species in a single request: *Mycoplasma bovis*-positive swabs (cattle), *Mycoplasma hyopneumoniae*-positive tissue samples from finishing pigs, and Infectious Bronchitis QX-positive samples from broiler poultry.

## 3 Results

### 3.1 Centralized vs. federated query performance

Table 4 reports mean query times: centralized access completed in 20.2 ± 4.1 s, while federated access completed in 30.0 ± 2.2 s, making federated access 1.49 times slower (Figure 5). A two-sample Welch’s t-test confirmed that the difference between centralized and federated query times was statistically significant (*p* = 0.003). Because the data format, query, and underlying stored dataset were held constant, this overhead stems from federation itself, network roundtrips to five separate Pod endpoints, multi-source query planning, and joins across results from multiple endpoints rather than a single in-memory dataset. Centralized querying thus significantly outperforms federated querying in terms of data processing time. However, this comes at the expense of flexibility and in-place access. Federated querying introduces performance overhead but enables in-place access to distributed data sources, which is an essential capability in collaborative, multi-institutional settings. Both centralized and federated queries returned identical result sets (569 records), confirming query correctness across architectures.

**Table 4:** Centralized vs. federated access (cattle only).

Metric	Centralized access	Federated access
Data format	RDF/Turtle (graph-based)	RDF/Turtle (graph-based)
Total RDF triples (stored)	740,056 RDF triples	740,056 RDF triples (same 5 labs data, distributed across 5 pods)
Data sources	1 aggregated RDF source	5 solid pods
Storage topology	Single aggregated central file store	Five lab-controlled solid pods
Data protection	SHA-256 anonymization of sensitive fields	SHA-256 anonymization of sensitive fields
Access control	Repository permissions	W3C access control list (ACL)
Execution environment	Azure app service standard S2 (2 cores, 3.5 GB RAM)	Azure app service standard S2 (2 cores, 3.5 GB RAM)
Rows returned	569 records	569 records
Total query time	20.2 ± 4.1 s	30.0 ± 2.2 s

**Figure 5:**
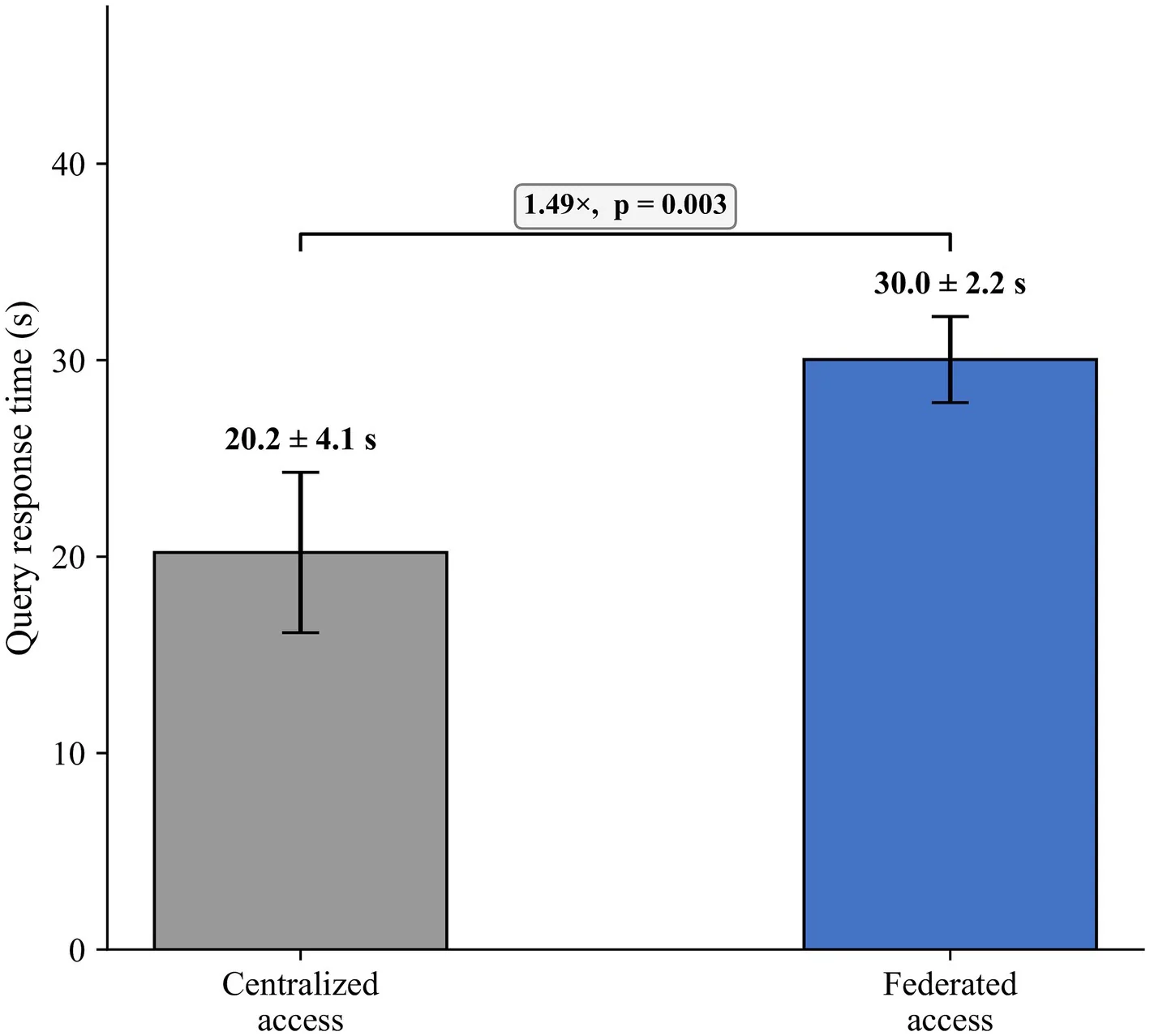
Centralized vs. federated query response time comparison.

### 3.2 Vertical vs. horizontal federation

As shown in Table 5 and Figure 6, the total number of RDF triples are 807,221 and reduces to 806,025 once processed with a query, a 1,196-triple gap caused by shared ontology statements (e.g., pathogen and breed type declarations) that each pod’s export embeds independently. Because an RDF graph and any triple that is identical across pods is represented only once, regardless of whether it is matched by the query’s filter conditions. A federated query merges the pods into a single RDF graph, so identical statements are counted once.

**Table 5:** Vertical (species-specific) and horizontal (mixed species) experiment results.

Metric	Vertical federation	Horizontal federation
Federation type	Species-specific pods	Mixed-species pods
Number of sources	12 pods	6 pods
Pod structure	5 cattle + 6 pig + 1 poultry	6 mixed (cattle + pig + poultry)
SPARQL query	Q2 (complex UNION)	Q2 (complex UNION)
Total RDF triples (stored)	807,221	807,221
Total RDF triples processed	806,025	806,025
Rows returned	1,378 (569 cattle, 59 pigs, 750 poultry)	1,378 (569 cattle, 59 pigs, 750 poultry)
Mean query time	30.1 ± 1.6 s	34.1 ± 3.4 s

The 1,196-triple difference between stored and processed triple counts reflects shared ontology-level statements (e.g., pathogen and breed type declarations) that each lab’s export embeds independently; federated querying treats the pooled pods as a single RDF graph, in which identical triples are represented once.

**Figure 6:**
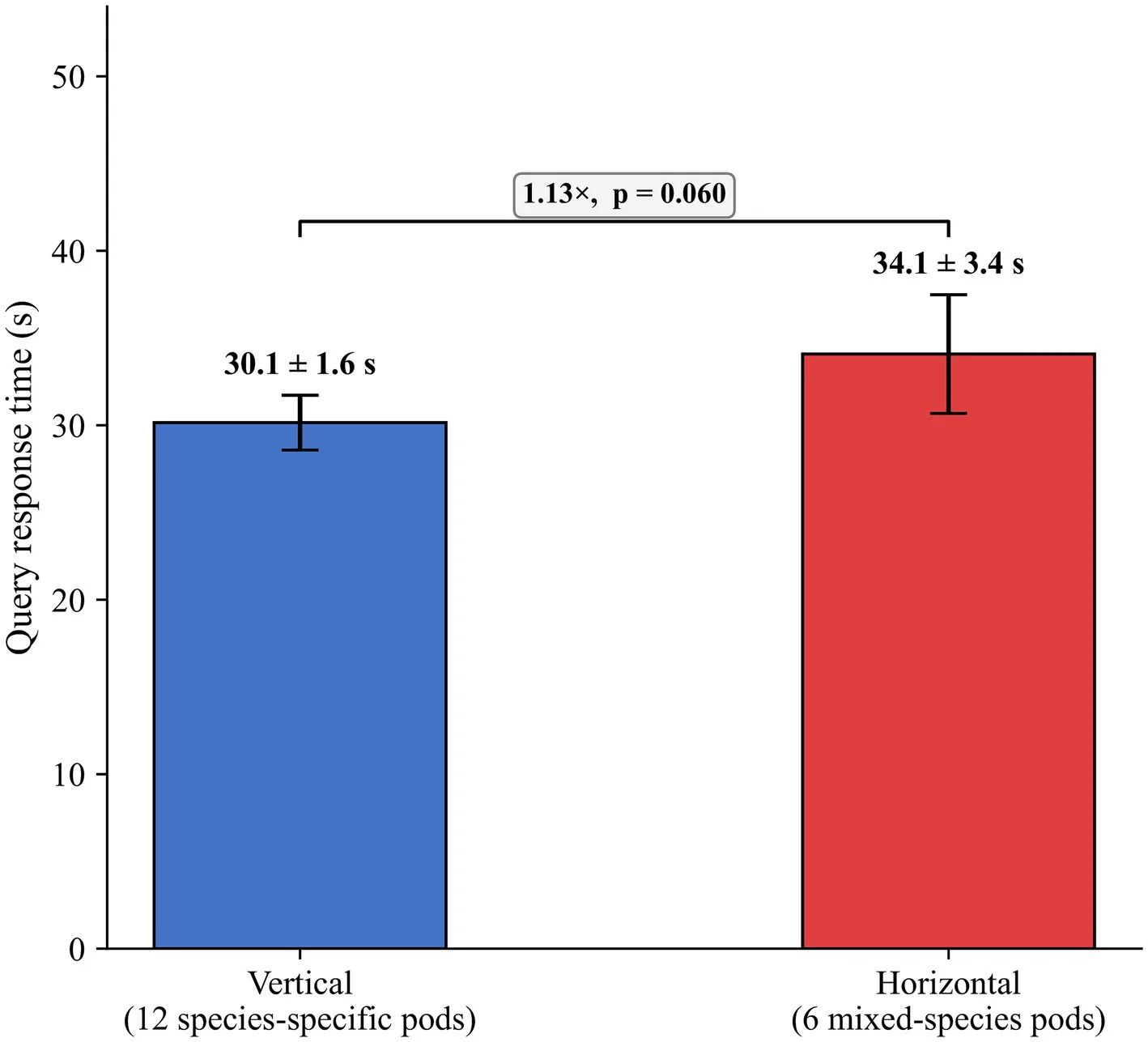
Vertical vs. horizontal federation performance comparison.

Vertical federation (12 species-specific Pods) completed in 30.1 ± 1.6 seconds. Horizontal federation (6 mixed-species Pods) completed in 34.1 ± 3.4 s. A Welch’s *t*-test indicated no statistically significant difference between vertical and horizontal configurations (*p* = 0.060); the borderline *p*-value reflects the modest sample size (*n* = 5), and the practical interpretation remains that the two configurations perform comparably. Horizontal federation was only 1.13 times slower than vertical federation, a small difference. Both configurations returned identical results (1,378 records: 569 cattle, 59 pigs, 750 poultry), confirming that the federation strategy does not affect query correctness. Because the vertical configuration used 12 species-specific Pods and the horizontal configuration 6 mixed-species Pods, the federation design and the number of sources differ. This comparison, therefore, reflects two realistic deployment configurations rather than an isolated effect of the federation strategy, and the small difference cannot be attributed solely to the vertical-versus-horizontal design.

### 3.3 Comparative summary across operational criteria and FAIR maturity

The three approaches show distinct profiles when characterized across the seven operational criteria defined in section 2.3. Table 6 presents a comparative summary of three data access approaches; the bottom row reports the FAIR maturity of the representative data artifact each approach publishes, scored against the 41 indicators of the RDA FAIR Data Maturity Model. Across the seven operational criteria, direct data sharing is anchored to its simplicity (high data access, high ease of use) but trades that simplicity for low workflow scalability, weak privacy controls, and limited consistency, and produces data artifacts with the lowest FAIR maturity in this study (6%). Centralized access uses an automated pipeline that keeps the data consistent and produces a data artifact with substantially higher FAIR maturity (60%). The trade-offs are its dependence on a single server and central custody of the data. Federated access keeps data under each laboratory’s control via per-source ACLs and an ontology-aligned RDF representation, delivering the highest workflow scalability and the highest FAIR maturity of the published data artifact in this study (83%), but at the highest implementation cost.

**Table 6:** Comparative summary of the three data access approaches across seven operational criteria and FAIR maturity.

Evaluation criteria		Direct data sharing	Centralized access	Federated access (solid pods)
Data access mechanism		High	Moderate	High
Privacy and governance		Low	Moderate	High
Integration workflow		Low	Moderate	High
Data consistency		Low	High	High
Workflow scalability		Low	Moderate	High
Ease of use and implementation		High	Moderate	Moderate
TCO	Personnel	High	Moderate	Moderate
	Infrastructure	Low	Moderate to high	Moderate to high
	Maintenance	High	Moderate	Moderate to high
FAIR maturity (S3)		6%	60%	83%

Ratings are qualitative (High/Moderate/Low) based on seven evaluation criteria in the Cattle Barometer case study. TCO, total cost of ownership (personnel, infrastructure, maintenance). Performance refers to query execution speed and system responsiveness and does not apply to direct sharing because data is exchanged manually. Ratings for the seven operational criteria are qualitative (High/Moderate/Low), based on the Cattle Barometer case study. The FAIR maturity row is quantitative: the percentage of the 41 RDA FAIR data maturity model indicators met by each approach’s published data (per-indicator scoring in Supplementary Data Sheet S3).

## 4 Discussion

This study found that centralized access completed queries significantly faster than federated access (20.2 ± 4.1 s vs. 30.0 ± 2.2 s, *p* = 0.003), while vertical and horizontal federation performed comparably. FAIR maturity of the published data increased from 6% under direct sharing to 60% under centralized access and 83% under federated access. These findings are discussed below in relation to each approach’s practical trade-offs and the existing literature.

Direct data sharing remains useful for infrequent short-term or low-volume exchanges, particularly in early project stages where simplicity matters more than technical overhead. Although FAIRness is a property of the data itself, its preservation depends heavily on the sharing method. In direct sharing, even data that initially follows FAIR principles can lose these qualities during informal exchanges. Metadata, schema definitions, and version histories are rarely transferred, especially when files are shared via email or private folders without centralized tracking. Over time, this FAIRness is hard to sustain. Version control tools could help, but the time and effort required to apply it consistently grows with every new file and update, so it is difficult to sustain in practice. The issue is not that the data is inherently non-FAIR, but that direct sharing lacks the mechanisms to preserve or promote FAIRness over time. This limitation is particularly visible in multi-stakeholder settings, for example the current use case where data exchange depends on coordination between different labs, file versions, and analysts. From the TCO perspective, infrastructure costs are low, but repeated manual effort for cleaning, merging, and maintenance makes personnel costs high. Over time, this approach becomes resource-intensive for larger or long-term deployments.

Centralized data sharing is best suited for coordinated workflows requiring standardized formats, automation, and high-performance data pipelines, making it ideal for surveillance dashboards and routine processing. Privacy is moderately protected through techniques like anonymization of sensitive data. This prevents reverse engineering but also means original identifiers cannot be recovered later, even under secure conditions. As a result, data may lose value for users or systems requiring linkage to sources, and anonymization can reduce interoperability if identifiers cannot be aligned across datasets or domains. TCO is moderate to high: setup requires time, technical expertise, and infrastructure, but once established, automated pipelines reduce daily effort. Tools like Tableau^8^ or R Shiny^9^ can be used to present the processed outputs in a user-friendly way, making it easy to explore the data without needing to understand the technical backend. This model works when providers accept centralized control and consistently supply data in agreed formats.

Federated access provides the greatest privacy, flexibility, and semantic interoperability. It is best for independent data providers who prioritize data ownership and selective access. Data remains under full control of providers, and in-place querying is enabled through a federated SPARQL engine. However, once authenticated access is granted, users can still copy the results they receive, meaning that governance is still required to regulate downstream use. Integration is achieved dynamically via shared ontologies, and new data sources can be added flexibly. Ease of use and implementation in this model differ by role; technically, it requires familiarity with Pods, RDF, SPARQL, and access control policies, making setup demanding. Yet, like centralized access, the final outputs can be consumed by end-users through a friendly interface, where backend complexity is hidden. TCO is high because each server requires hosting, updates, and technical oversight, even when automation reduces daily work. Despite higher costs, federated access supports long-term scalability and modularity, especially in privacy-sensitive or multi-institutional environments. Although more expensive, federated access systems strike a balance between sovereignty and interoperability. Their cost–benefit profile must be weighed carefully before production-scale deployment. This model suits distributed networks such as independent labs or stakeholders, but only if users are trained in ontologies and understand their data quality and responsibilities. In practice, this is a major limitation: metadata are often incomplete, and stakeholders struggle to document datasets (3, 20). Centralized access faces similar hurdles, requiring major effort for metadata, schema alignment, and ingestion pipelines. The Cattle Barometer demonstrates that centralized or federated access is technically feasible and provides a solid foundation for future refinement and scaling.

Across the three approaches, FAIR maturity ranged from 6% for direct sharing, through 60% for centralized access, to 83% for federated access. As described in Section 2.1-2.2, the data artifacts produced by the three approaches all originate from the same underlying data source, which was progressively cleaned, harmonized, and ontology-mapped as it moved from direct to centralized and federated access. The FAIR maturity gradient therefore, partly reflects this evolution in data management and cannot be attributed to the access approach alone; the enrichment steps that raise FAIRness are, however, themselves afforded by the centralized and federated architectures and structurally unavailable in direct sharing. As detailed in Supplementary Data Sheet S2, federated access scores higher on interoperability and reusability because each lab’s data is published as directly retrievable, ontology-aligned RDF resources. In contrast, in the centralized approach, the equivalent RDF representation remains internal and is not exposed as individually addressable linked data. This suggests that achieving high FAIR maturity depends less on the specific access architecture chosen than on whether FAIR-by-design practices, such as ontology alignment and machine-actionable publishing, are deliberately embedded in the data workflow, regardless of approach.

The architectural trade-offs we observe also appear in similar studies from human-health research. Rujano et al. (19) compared centralized and federated approaches across five large European life-sciences use cases, and found that centralized models make it easier to link and harmonize data. In contrast, federated models make it easier to scale up and remain compliant with data protection rules because the data stay with the original owner. These are the same trade-offs we see in livestock surveillance. Welten et al. (18) showed multi-institutional secure onboarding for distributed analytics in clinical research, and Sinaci et al. (20) extended the federated approach to training machine-learning models on FAIR health data. Choudhury et al. (34) went further and applied federated learning to distributed health records. Our work extends this body of evidence from human health to animal health by using a similar federated architecture (Solid Pods + federated SPARQL) on livestock disease data, delivering high FAIR maturity for the published data artifacts (83%), with a modest but statistically significant query-latency overhead of 1.49 × over centralized access, though, as discussed in Limitations, this single-server setup likely underestimates the overhead of genuinely distributed, cross-country federation. For animal-health surveillance in practice, the access architecture directly shapes how quickly and how widely diagnostic signals can be turned into action: federated access preserves laboratory data sovereignty while still enabling cross-border querying on which early detection and coordinated One Health response depend, whereas centralized access favors high-performance routine dashboards built on already consolidated data.

Within agriculture, recent work by Krisnawijaya et al. (4) across dairy and fish-farming projects shows that FAIR principles can be put into practice when secure access protocols, semantic interoperability, and clear metadata standards are designed in from the start. Delavenne et al. (6) assessed 30 European datasets with animal health data used in a research project and found that the rare use of metadata and standard vocabularies is a major gap. The same research group has subsequently introduced the first community-developed rich-metadata guidelines for veterinary epidemiology datasets (35), positioned at the early steps of the FAIR continuum and referencing ontology-driven approaches such as the Livestock Health Ontology, as used in the Cattle Barometer evaluated here, as the natural next step. They are paving the way to create community-specific guidelines toward the longer-term shift toward FAIR-by-design pipelines advocated by Candela et al. (36).

Federated SPARQL querying over Solid Pods worked reliably in our experiments after we fixed a reliability issue in the underlying engine. Technology is still maturing, and the wider community is actively improving its performance and reliability, including through this work. We ran into query failures at larger data volumes under earlier Comunica versions, a problem typical at this stage of the ecosystem’s development. Working with the Comunica development team, we helped test and validate a query-planning fix that resolved it; all experiments reported here use the corrected version (Comunica v5.2.0). Surveillance programs, considering a federated approach, should budget for this kind of ongoing engagement with the underlying tooling as part of total cost of ownership, alongside initial setup. It is also worth noting that the automated pipelines described here already implement several DataOps practices: scripted, version-controlled ingestion and processing via GitHub, and reproducible outputs archived publicly (GitHub, Zenodo). However, the ontology-mapping step depends on incoming data matching a specific expected format and fails without graceful handling when it does not; the pipelines do not yet extend to continuous data-quality monitoring, automated alerting on pipeline failures, schema validation, or automated testing of the mapping logic itself, as advocated by the broader DataOps paradigm (37). Incorporating these additional practices, alongside structured collaboration features between data providers and analysts, would support centralized and federated surveillance pipelines’ reliable operation and maintenance at scale as more laboratories are onboarded. DataOps paradigm (37)also situates the investigation of business value as part of the DataOps discipline; for the Cattle Barometer specifically, this dimension is addressed separately in the DECIDE project’s exploitation planning, which identifies sustainable hosting and revenue-stream challenges for maintaining non-commercial surveillance tools beyond the project’s funding period (38).

This study has some limitations. The current experiments used a limited number of Pods (5 to 12) hosted on a single Solid server, so performance may differ when scaling to a larger number of geographically distributed Pods across multiple servers. The current experiments used a limited number of pods (5 to 12) hosted on a single Solid server, so this study does not capture the network latency, firewalls, and heterogeneous infrastructure of genuinely cross-institutional federation, where laboratories are geographically distributed across different countries; real-world federated performance in such a deployment could be considerably worse than the 1.49 × overhead reported here. In addition, the two federation configurations did not use the same number of Pods (12 vertical vs. 6 horizontal), so federation design and source count varied together; future work should compare vertical and horizontal federation at matched pod counts to isolate the effect of federation strategy from that of source count. Because the three data artifacts compared here originate from a single pipeline in which each subsequent access approach receives cumulatively enriched data (direct sharing draws on the least-processed data, federated access on the most), federated access’s FAIR maturity advantage may partly reflect its position at the end of this enrichment pipeline by construction, rather than an inherent property of the federated architecture alone. Future work could disentangle this by applying an equivalent enrichment pipeline independently to each access approach before comparison.

Distributed-SPARQL optimization techniques such as result-aware source selection (39), and SPARQL result caching with pod-granularity tuning (40) could help reduce query time at that scale. Additionally, rather than running an automated tool ourselves, such as AgReFed (41) or F-UJI (42), we manually scored each approach against the standardized 41 indicators of the RDA FAIR Data Maturity Model (25) and reported transparent per-indicator notes. This manual, single-framework choice avoids the tool-to-tool disagreement documented by Krans et al. (43) and Candela et al. (36), who identified inconsistencies in the assessment results across identical datasets. While Candela et al. (36) advocate for using community-specific assessment tools, the absence of such tools for veterinary epidemiology data made scoring against the established RDA framework the most practical approach. However, this manual, localized evaluation may make it harder to compare our results with future automated studies directly.

## 5 Conclusion

This study compares direct, centralized, and federated data access approaches in veterinary surveillance, using the Cattle Barometer as a real-world testbed. Centralized access completed the benchmark query in 20.2 ± 4.1 s versus 30.0 ± 2.2 s for federated access, a statistically significant 1.49 × overhead. Vertical and horizontal federation strategies returned identical results (1,378 records: 569 cattle, 59 pigs, 750 poultry) and performed comparably, though the two configurations used a different number of Pods (12 vs. 6). The FAIR maturity of the published data artifacts was 6% for direct sharing, 60% for centralized access, and 83% for federated access. The choice between centralized and federated access is therefore a trade-off between query speed and data sovereignty. Centralized access suits high-performance dashboards on harmonized data, while federated access suits multi-laboratory settings where data must remain with the original owner. The federated access approach can be extended in future work toward privacy-preserving distributed model training.

## Data Availability

The diagnostic datasets used in this study are sensitive and subject to data-sharing agreements with participating laboratories. Access to the original data may be provided on reasonable request and with appropriate approvals. To support reproducibility, the code, Jupyter notebook, and sample RDF dataset are openly available via Zenodo: https://zenodo.org/records/21825431.

## Footnotes

1
https://solidserver.bovi-analytics.com

2
https://comunica.dev/

3
https://decide-federated-functions.azurewebsites.net/api/FedQuery

4
https://decide-project-eu.github.io/case-studies-website/tutorials/cattle-barometer.html

5
https://github.com/decide-project-eu/case-studies-website

6
https://github.com/djsf-kobayashi/penny

7
https://github.com/decide-project-eu/data-access-guidelines-best-practices-wp1-decide

8
https://decide-project-eu.github.io/case-studies-website/case-studies/cattle-barometer.html

9
https://connect.posit.vetinst.no/laksetap/

## Supplementary material

SUPPLEMENTARY DATA SHEET S1 Characterization of the three data access approaches across seven operational criteria (data access, privacy and governance, integration workflow, consistency, workflow scalability, ease of use, and total cost of ownership).

SUPPLEMENTARY DATA SHEET S2 Qualitative analysis of how each data access approach addresses the four FAIR principles (findability, accessibility, interoperability, reusability), explaining the factors underlying the maturity scores in S3.

SUPPLEMENTARY DATA SHEET S3 FAIR data maturity scoring: per-indicator assessment of each approach’s published data artifact against the 41 indicators of the RDA FAIR data maturity model.

## References

[1] Tegegne HA Freeth FT Bogaardt C Taylor E Reinhardt J Collineau L . Implementation of one health surveillance systems: opportunities and challenges-lessons learned from the OH-EpiCap application. One Health. (2024) 18:100704. doi: 10.1016/j.onehlt.2024.100704, 38496337 PMC10940803

[2] Benedetti G Boisen N Prada JM. One health surveillance in practice: experiences of integration among human health, animal health, environmental health, and food safety sectors. Front Public Health. (2024) 12:1384988. doi: 10.3389/fpubh.2024.1384988 PMC10940499 38496385

[3] Ahoa E Kassahun A Verdouw C Tekinerdogan B. Challenges and solution directions for the integration of smart information systems in the agri-food sector. Sensors. (2025) 25:2362. doi: 10.3390/s25082362, 40285052 PMC12031551

[4] Krisnawijaya NNK Tekinerdogan B Catal C van der Tol R Herdiyeni Y. Implementing FAIR principles in data management systems: a multi-case study in precision farming. Comput Electron Agric. (2025) 230:109855. doi: 10.1016/j.compag.2024.109855

[5] Brunori G Bacco M Puerta-Piñero C Borzacchiello MT Stormer E. Agri-food data spaces: highlighting the need for a farm-centered strategy. Data Brief. (2025) 59:111388. doi: 10.1016/j.dib.2025.111388, 40124292 PMC11928818

[6] Delavenne C van Schaik G Frössling J Cameron A Faverjon C. Reusability challenges of livestock production data to improve animal health. Sci Data. (2025) 12:458. doi: 10.1038/s41597-025-04785-4, 40108251 PMC11923216

[7] Noor S Degroote J van Schaik G Pardon B Faverjon C Delavenne C . A systematic review on the role of livestock ontologies in animal health management and disease surveillance: a PRISMA 2020 and AI-assisted screening approach. Smart Agric Technol. (2026) 14:101977. doi: 10.1016/j.atech.2026.101977

[8] Raineri RR Shivley CB Plummer PJ Kreuder AJ. Assessing stakeholders’ opinions on sharing antimicrobial susceptibility testing data from animals is key to developing a centralized database and dashboard tool. J Am Vet Med Assoc. (2024) 262:216–25. doi: 10.2460/javma.23.07.0426, 37944260

[9] Dijkstra E Vellema P Peterson K Bogt-Kappert C ter Dijkman R Harkema L . Monitoring and surveillance of small ruminant health in the Netherlands. Pathogens. (2022) 11:635. doi: 10.3390/pathogens11060635 PMC9230677 35745489

[10] Santman-Berends IMGA Brouwer-Middelesch H Van Wuijckhuise L De Bont-Smolenaars AJG Van Schaik G. Surveillance of cattle health in the Netherlands: monitoring trends and developments using routinely collected cattle census data. Prev Vet Med. (2016) 134:103–12. doi: 10.1016/j.prevetmed.2016.10.002, 27836031

[11] Dupuy C Locquet C Brard C Dommergues L Faure E Gache K . The French national animal health surveillance platform: an innovative, cross-sector collaboration to improve surveillance system efficiency in France and a tangible example of the one health approach. Front Vet Sci. (2024) 11:1249925. doi: 10.3389/fvets.2024.1249925, 39234170 PMC11371667

[12] Bokma J. Santman-Berends I. Vidal G. Hostens M. Ribbens S. Evrard J. European veterinary barometer for bovine respiratory diseases: a tool showing diagnostic test results and geolocation of respiratory tract samples from cattle Proceedings of the 2023 European Buiatrics Congress and ECBHM Jubilee Symposium (2023) Ghent: Ghent University

[13] Bokma J Noor S Pardon B Hostens M. Cattle barometer. DECIDE Case Stud. (2023). doi: 10.5281/zenodo.13354666

[14] Meyer A Faverjon C Hostens M Stegeman A Cameron A. Systematic review of the status of veterinary epidemiological research in two species regarding the FAIR guiding principles. BMC Vet Res. (2021) 17:270. doi: 10.1186/s12917-021-02971-1, 34380468 PMC8355576

[15] Houe H Nielsen SS Nielsen LR Ethelberg S Mølbak K. Opportunities for improved disease surveillance and control by use of integrated data on animal and human health. Front Vet Sci. (2019) 6:301. doi: 10.3389/fvets.2019.00301, 31572735 PMC6753377

[16] Dedecker R. Slabbinck W. Wright J. Hochstenbach P. Colpaert P. Verborgh R. What’s in a pod? A knowledge graph interpretation for the solid ecosystem. Proceedings of the 6th Workshop on Storing, Querying and Benchmarking Knowledge Graphs (QuWeDa) at ISWC 2022 (2022)

[17] Sambra A. V. Mansour E. Hawke S. Zereba M. Greco N. Ghanem A. (2016) Solid: a platform for decentralized social applications based on linked data MIT CSAIL Qatar Comput Res Inst Tech Rep. Available online at: https://cs.brown.edu/courses/csci2390/2021/readings/solid.pdf (Accessed July 9, 2026).

[18] Welten S Hempel L Abedi M Mou Y Jaberansary M Neumann L . Multi-institutional breast cancer detection using a secure on-boarding service for distributed analytics. Appl Sci. (2022) 12:4336. doi: 10.3390/app12094336

[19] Rujano MA Boiten J-W Ohmann C Canham S Contrino S David R . Sharing sensitive data in life sciences: an overview of centralized and federated approaches. Brief Bioinform. (2024) 25:bbae262. doi: 10.1093/bib/bbae262, 38836701 PMC11151787

[20] Sinaci AA Gencturk M Alvarez-Romero C Erturkmen GBL Martinez-Garcia A Escalona-Cuaresma MJ . Privacy-preserving federated machine learning on FAIR health data: a real-world application. Comput Struct Biotechnol J. (2024) 24:136–45. doi: 10.1016/j.csbj.2024.02.014, 38434250 PMC10904920

[21] Ghafoor A Symeonidis I Rydberg A Lindahl C Abbasi AQ. Towards empowering stakeholders through decentralized trust and secure livestock data sharing. Cryptography. (2025) 9:52. doi: 10.3390/cryptography9030052

[22] Ganesan LP Krishnan S. Blockchain-enabled, nature-inspired federated learning for cattle health monitoring. Informatics. (2025) 12:57. doi: 10.3390/informatics12030057

[23] Wilkinson MD Dumontier M Aalbersberg I Appleton G Axton M Baak A . The FAIR guiding principles for scientific data management and stewardship. Sci Data. (2016) 3:18. doi: 10.1038/sdata.2016.18 PMC4792175 26978244

[24] Wilkinson MD Dumontier M Sansone S-A Bonino da Silva Santos LO Prieto M Batista D . Evaluating FAIR maturity through a scalable, automated, community-governed framework. Sci Data. (2019) 6:174. doi: 10.1038/s41597-019-0184-5, 31541130 PMC6754447

[25] Bahim C Casorrán-Amilburu C Dekkers M Herczog E Loozen N Repanas K . The FAIR data maturity model: an approach to harmonise FAIR assessments. Data Sci J. (2020) 19:41. doi: 10.5334/dsj-2020-041

[26] van Schaik G Hostens M Faverjon C Jensen DB Kristensen AR Ezanno P . The DECIDE project: from surveillance data to decision-support for farmers and veterinarians. Open Res Eur. (2023) 3:82. doi: 10.12688/openreseurope.15988.2 PMC11109551 38778904

[27] Garcia-Morante B. Introducing the pig barometer: a comprehensive pilot tool for mapping diagnostic test results and geolocation of swine endemic pathogens in Spain Proceedings of the 2025 European Symposium of Porcine Health Management (2025)

[28] Bokma J Doidge C Jourquin S Hostens M Noor S Santman-Berends I . The DECIDE cattle barometer: a cross-country monitoring and decision support tool for respiratory pathogens. Ir Vet J. (2026). doi: 10.1186/s13620-026-00356-0

[29] Noor S. decide-project-eu/LiveStockHealthOntologyWp1: Initial Release. Switzerland: Zenodo. (2023)

[30] Noor S Bokma J Pardon B Van Schaik G Hostens M. "Agri semantics: developments to improve data interoperability to support farm information management and decision support systems in agriculture". In: Sørensen CG, editor. Smart Farms: Improving Data-Driven Decision Making in Agriculture. Burleigh: Dodds Science Publishing (2024). p. 75–96.

[31] Noor S Degroote J Van Schaik G Pardon B Bokma J Slegers Y Advancing precision livestock farming through ontology-driven interoperable health data management: extending the livestock health ontology (LHO) for enhanced disease surveillance Proceedings of the 11th European Conference on Precision Livestock Farming (ECPLF) (2024)

[32] Taelman R Verborgh R Link traversal query processing over decentralized environments with structural assumptions Proceedings of the 2023 International Semantic Web Conference Berlin: Springer (2023)

[33] Taelman R. Van Herwegen J. Vander Sande M. Verborgh R. Comunica: a modular SPARQL query engine for the web Proceedings of the 2018 International Semantic Web Conference Berlin: Springer (2018) doi: 10.1007/978-3-030-00668-6_15

[34] Choudhury O. Park Y. Salonidis T. Gkoulalas-Divanis A. Sylla I. k Das A. Predicting adverse drug reactions on distributed health data using federated learning Proceedings of the 2020 AMIA Annual Symposium (2020) PMC7153050 32308824

[35] Faverjon C Delavenne C Bareille S Cameron A Carmo LP Dean KR . Enhancing reusability of veterinary epidemiological data by creating contextual metadata. Res Sq. (2026). doi: 10.1038/s41597-026-08217-9

[36] Candela L Mangione D Pavone G. The FAIR assessment conundrum: reflections on tools and metrics. Data Sci J. (2024) 23:33. doi: 10.5334/dsj-2024-033

[37] Ereth J. (2018) DataOps-towards a definition. Available online at: http://ceur-ws.org/Vol-2191/paper13.pdf (Accessed July 9, 2026).

[38] Berends S Ing Veldhuis A Wouter S Mirjam N (2025) Roadmap for exploitation. [DECIDE project deliverable]. DECIDE project (Horizon 2020, grant agreement no. 101000494). Available online at: https://decideproject.eu/wp-content/uploads/sites/41/2026/07/DECIDE_D6.8_Roadmap_for_Exploitation.pdf (Accessed July 9, 2026).

[39] Aimonier-Davat J Nédelec B Dang M-H Molli P Skaf-Molli H FedUP: querying large-scale federations of SPARQL endpoints. Proceedings of the ACM Web Conference 2024 New York, NY: ACM (2024)

[40] Peng P Zou L Özsu MT Chen L Zhao D. Processing SPARQL queries over distributed RDF graphs. VLDB J. (2016) 25:243–68. doi: 10.1007/s00778-015-0415-0

[41] Bahlo C. Advancing FAIR agricultural data: the AgReFed FAIR assessment tool. Data Sci J. (2024) 23:33. doi: 10.5334/dsj-2024-018

[42] Petrosyan L Aleixandre-Benavent R Peset F Valderrama-Zurián JC Ferrer-Sapena A Sixto-Costoya A. FAIR degree assessment in agriculture datasets using the F-UJI tool. Ecol Inform. (2023) 76:102126. doi: 10.1016/j.ecoinf.2023.102126

[43] Krans N Ammar A Nymark P Willighagen E Bakker M Quik J. FAIR assessment tools: evaluating use and performance. NanoImpact. (2022) 27:100402. doi: 10.1016/j.impact.2022.100402, 35717894

