# Supplementary material for: From data silos to actionable insights: a comparative evaluation of data access models and practical guidelines for animal health surveillance
Source: Front Vet Sci. 2026 Sep 17;13:1962800. doi: 10.3389/fvets.2026.1962800 (PMC13631730; doi:10.3389/fvets.2026.1962800)
Supplement: Supplementary file 1 [file Data_Sheet_1.DOCX]

**Supplementary Material S1**

### 1. **Characterisation of Direct data sharing**

**Data access mechanism:** One of the main advantages of direct data sharing is its simplicity in terms of data access. It is easy, quick, and cheap to set up, as it requires no complex infrastructure or integration platforms to start sharing and accessing data. Data providers can participate by simply exporting data in spreadsheet formats and sharing it via common channels such as email or cloud folders. From a data sharing agreement perspective, this approach is also quite straightforward, as it involves only bilateral agreements, which are usually easier to set up. The simplicity of the approach comes at the cost of time. Indeed, the absence of automatic steps to get access to the data can make this task time-consuming for both the data providers and the data analysts.

**Data privacy and governance**: Data analysts can end up being the sole guardians of data privacy if they are also in charge of data anonymization. When done manually on an ad hoc basis, it can be associated with errors or inconsistencies and therefore with risks for data privacy.

**Data integration workflow**: In this setup, the burden of data cleaning and integration usually relies mostly or only on the data analyst. It has advantages for the data provider who does not need to invest much before making their data accessible to the data analyst (e.g., no specific data format is needed, no specific infrastructure either). However, it comes with challenges for the data analyst because data cleaning and integration of heterogeneous data can be very complex and quickly become time-consuming. And if the data provider later changes the format or structure, the maintenance effort increases even further.

**Data consistency**: Direct sharing is prone to human error and versioning problems, as manually cleaned static files must be updated and re-shared without real-time synchronization. This causes risks in terms of data consistency, as each new data upload may not be consistent with the previous one.

**Workflow scalability:** Direct data sharing scales poorly because the effort grows linearly with the number of providers. As the number of data providers increases, so does the variety of data formats, schemas, and quality levels, each requiring individualized cleaning and harmonization. This not only increases the workload for analysts but also introduces bottlenecks, especially when updates are frequent or time-sensitive. For example, in the Cattle Barometer use case, integrating CSV files from multiple labs required repeating anonymization and transformation steps manually for each new file submission. Moreover, the absence of automated data pipelines means there is no efficient way to monitor new incoming data, validate files, or update visualizations in real-time. Instead, each step, requesting data, downloading, cleaning, merging, and uploading, is repeated manually, making it infeasible for continuous data streams or for scaling to dozens of labs or species. This results in delays, inconsistent update cycles, and significant overhead coordination, which makes the model unsuitable for long-term or large-scale surveillance systems.

**Ease of use and implementation**: For data providers, direct sharing is simple and requires no technical setup. However, handling and sending files manually again and again can become tiring over time. For analysts, the diversity of data formats used means extra work is needed to clean and prepare the data each time it is received.

**TCO for direct data sharing**: In a direct data sharing setup, most of the cost comes from personnel time. Since data is shared manually, people must spend time cleaning, merging, and interpreting files every time something changes. Infrastructure needs are low because basic tools like email or cloud folders are enough. However, the lack of automation means staff must repeat the same work for every new dataset. Maintenance is also high, since there’s no central system to keep things organized or updated automatically. This dependency on individuals also makes the process fragile; when staff leave or change roles, then knowledge about file structures and data handling is lost.

## **2. Characterisation of centralized access**

**Data access:** In centralized access, providers transfer their data to a central system where automated pipelines reduce workload for both providers and analysts. While convenient, this setup requires substantial technical implementation and raises concerns about privacy and user autonomy [18], making legal agreements essential. In the Cattle Barometer, access was governed by bilateral contracts with participating laboratories under the DECIDE consortium framework.

**Data privacy:** Centralized access streamlines processing but raises concerns about data sovereignty and misuse, particularly in multi-institutional or cross-border contexts. Mitigation requires strong governance, access control, and legal agreements. In the Cattle Barometer, regional aggregation and SHA-256 anonymization reduced privacy risks, though these measures also limited reusability and interoperability, underscoring trade-offs between protection and long-term utility.

**Data integration:** Centralized data access automates data cleaning and integration, significantly reducing human error and the workload for analysts. It also facilitates data reuse since all information is stored in one place. However, this requires that incoming data follow a predefined structure, something often lacking in veterinary datasets [3]. In addition, the central platform creates infrastructure dependency, acting as a single point of failure and requiring costly maintenance.

**Data consistency**: The centralized model offers strong consistency benefits. All data passes through uniform scripts and is mapped to a standardized schema before storage, ensuring harmonized structure and terminology across labs. The automated ingestion process eliminates manual merging, reducing errors and ensuring stable, version-controlled datasets suitable for longitudinal analysis.

**Workflow scalability:** Centralized systems offer moderate scalability. New labs can be added to the pipeline, but only if they adhere to the predefined data format or if additional scripts are developed for their specific structure. This format dependency poses a bottleneck: while adding more data is technically feasible, variability in incoming file structures can limit the ability to scale efficiently. The system also relies on sufficient server resources to handle increasing data volume and update frequency.

**Ease of use and implementation:** Once the infrastructure is in place, the system is relatively easy to use for both data providers and analysts, assuming data is submitted in the expected format. Providers simply upload files to the central platform, and analysts receive clean, ready-to-visualize datasets via automated processing. However, setting up the system requires technical expertise in scripting, data transformation, and server management. Additionally, changes in data structure require manual intervention to update or extend the scripts, adding to long-term maintenance needs.

**TCO for Centralized Data Sharing**: In a centralized setup, the initial setup requires time and technical expertise to define data formats, develop processing scripts, configure cloud servers, and test dashboards. But once the system is running, personnel effort drops thanks to automation. Infrastructure costs (cloud storage, processing tools) remain ongoing, and some maintenance is needed to monitor performance and keep the pipeline updated. This model is more efficient over time, especially when data arrives in consistent formats.

### 3. **Characterisation of federated access (Federated via Bovi-Analytics PODS)**

**Data access:** Federated access via Bovi-Analytics PODS offers real-time querying without transferring ownership of data. However, even without transferring ownership, anyone with query access can still copy the data they receive, so control is maintained only at the access point. It means each laboratory maintains its own data on PODS, and authorized consumers can access it using a federated SPARQL query engine. This model enables live access to distributed data. Initial setup can be complex, but once in place, queries can be executed dynamically via the API. However, since authorized users can still copy the data they retrieve, some level of agreement or governance may still be necessary to regulate downstream use.

Each provider can define simple access conditions, such as who may query their data and under what constraints, through pod-level configuration or informal agreements. Table 1 shows that access is enforced via Web Access Control (WAC), which shows the read-only public policy for demo data and the full permissions retained by the owner.

**Data privacy**: Data privacy is a core strength of this model. Because each data provider retains full control of their Solid pod, they determine who can access what information, and under what conditions. Access control is enforced through W3C-compliant policies, minimizing governance risks. No data is centrally stored or duplicated, and sensitive information remains under the direct control of its owner. However, once a query is executed, the returned results can be copied or stored by the requester, meaning that duplication can occur outside the PODs and must be managed through governance rather than technical controls.

**Data integration:** Integration is achieved at query time through semantic alignment. All RDF datasets are structured using a shared ontology LHO, which enables consistent interpretation of data across different labs and species. However, unlike centralized access, where integration is done upfront, in federated access, integration is virtual and performed dynamically. This means integration is flexible and non-invasive, but it also requires well-aligned schemas and careful ontology design to avoid mismatches or ambiguity during queries.

**Workflow scalability:** This architecture is highly scalable. New labs or species can be added simply by linking new pod Uniform Resource Locators (URLs), without changing the core system. For example, when permission is granted, adding the lab’s Solid Pod URL (see Experiment 2) makes its data available. The federated query engine can dynamically discover and query multiple pods in parallel using the list of POD endpoints supplied by the POD owner, regardless of how many are connected.

**Ease of Use and Implementation:** The federated access using Solid Pods is powerful, but it can be challenging to use without technical help. Once the system is running, analysts can access data from different sources in real time, without needing to collect files manually. However, using it requires writing SPARQL queries, a special type of computer language that is used to query the federated data.

**TCO for Decentralized (Solid Pods) Federation**: Setting up a federated access involves significant effort at the start. Skilled technical staff or data analysts are needed to configure Solid Pods, deploy the SPARQL query engine, and ensure that all data follows shared ontologies. However, once the system is up and running, the amount of manual work decreases thanks to automation. Infrastructure costs remain higher than in other models because each lab or partner requires its own hosted pod and server setup. While automated pipelines help reduce repetitive tasks, ongoing maintenance is still complex. You need to make sure pods stay online, monitor query performance, and keep ontologies coordinated. This approach offers strong privacy and control, but it also requires more coordination, technical oversight, and long-term investment to keep everything working smoothly.
