# Supplementary material for: From data silos to actionable insights: a comparative evaluation of data access models and practical guidelines for animal health surveillance
Source: Front Vet Sci. 2026 Sep 17;13:1962800. doi: 10.3389/fvets.2026.1962800 (PMC13631730; doi:10.3389/fvets.2026.1962800)
Supplement: Supplementary file 2 [file Data_Sheet_2.DOCX]

**Supplementary Material S2**

Qualitative analysis of how each data access approach addresses the FAIR principles, explaining the factors underlying the maturity scores in S3. This analysis explains the FAIR maturity of the artifact each approach produces, from the perspective of external reuse. Because the underlying diagnostic data are sensitive, FAIR is applied as "as open as possible, as closed as necessary": the published metadata, code, and ontologies remain findable and reusable even where the data itself is access-controlled.

### 1.  **Direct data sharing approach**

**Data findability:** Data files such as “DECIDE_MTA_UGENT_14nov2022.xlsx”, and “DECIDE_xxLab_pig_diagnostic_data_for_ontologies_12102023.xlsx” were exchanged directly via email or shared drives. Although these files sometimes included structured schemas or explanatory notes, that contextual information was often lost during the file-sharing process. Key details such as schema definitions, descriptions, or version history were usually stored separately, for example, in emails or known only to individuals, and were not embedded within the files themselves. As a result, team members often struggled to identify the most recent version, leading to repeated requests and delays. The absence of a central location for storing or searching shared files made it difficult to trace what had already been exchanged. This lack of structure significantly reduced the findability of shared files across institutions. Critically, these files carry no persistent identifiers and are not registered in any indexed, searchable catalogue, so they cannot be discovered by anyone outside the direct exchange.

**Data accessibility:** In the Cattle Barometer use case, data accessibility can be obtained only by contacting the original sender; there is no standardized or automated retrieval protocol, no resolvable identifier, and no guarantee that metadata persists once the file is gone. Retrieval depends entirely on human intervention, which introduces delays and unpredictability.

**Data interoperability:** The interoperability of directly shared data is constrained by inconsistent formatting and the lack of enforced standards. Even when files are created with some structure, variations in naming conventions, column formats, or coding practices across contributors introduce semantic mismatches. Since there is no system to align schemas or ensure shared vocabularies, integrating data from multiple sources typically requires manual mapping and cleaning. This limits machine-readability and slows down scalable, automated integration of shared files and resources across systems.

**Data reusability:** Reusability is low because, even when data files are shared with documentation, metadata, and a clear structure, they are still hard to reuse later. This is because important information is often kept in separate places, like emails or notes, and not saved together with the file. Once the file is saved, copied, or changed, that extra information can be lost. There is usually no way to track updates or know where the data came from. This makes it difficult for others to understand or reuse the data later without asking the original sender again.

2. **Centralized access**

**Data findability:** Data stored centrally can, in principle, be catalogued and indexed, which would make datasets easier to locate. Dashboard integrations can further improve visibility for stakeholders. However, in the current DECIDE implementation, the centralized endpoint is not publicly exposed and sits behind firewalls, which limits external discoverability. Full findability benefits would require a public or authenticated metadata catalog and accessible endpoints.

**Data accessibility:** Unlike manual sharing models, in centralized access, the cleaned, aggregated dataset is retrievable over a standard web protocol (HTTPS) through the public Cattle Barometer dashboard and the project's public GitHub repository, where the R/Quarto processing pipeline is openly published. Retrieval can be automated (e.g., via GitHub raw access or the dashboard). The workflow code and the LHO ontology carry persistent Zenodo DOIs, but the dataset itself is not separately DOI-minted, and the original non-aggregated laboratory data remain behind institutional access controls.

**Data interoperability:** Interoperability is moderately supported. If the central system incorporates schema alignment or maps variables to shared ontologies during ingestion, semantic interoperability, the meaning and interpretation of the data is conserved. However, this depends on explicit design choices. Without semantic enrichment, the system remains syntactically interoperable (through consistent structure) but not semantically aligned across domains or species.

**Data reusability:** Cleaned and standardized datasets are highly reusable, especially when combined into a single harmonized output. Analysts can easily run additional queries or use the data in other tools without repeating the cleaning process. Reusability is maximized when the system includes metadata, data dictionaries, and persistent file storage.

3. **Federated access (Federated via Bovi-Analytics PODS)**

**Data findability:** In federated access using Solid Pods, data findability depends on how dataset URLs and metadata are structured, published, and made accessible. Solid Pods do not offer centralized indexing by default, so discoverability relies on explicit sharing, well-defined metadata, and standardized structuring. To improve discoverability, metadata from the schema.org vocabulary is embedded directly within each turtle file. Elements such as schema: Dataset, schema: name, schema: description, schema: creator, and schema:dateModified are now applied across all datasets. This makes each dataset more amenable to indexing by linked-data tools and semantic search. When combined with public or controlled-access sharing policies, this setup supports FAIR-aligned findability in a decentralized, federated environment. This can be found here: [solidserver.bovi-analytics.com/decide_lab1/Vertical/RDFoutputCattleSampleLab1.ttl](https://solidserver.bovi-analytics.com/decide_lab1/Vertical/RDFoutputCattleSampleLab1.ttl). However, findability is not yet maximal because the datasets are not registered in a central catalog or assigned a persistent identifier (DOI), which is reflected in the corresponding S3 score.

**Data accessibility:** In the federated setup, each dataset is dereferenceable by its URI over HTTPS and queryable through a SPARQL endpoint, with access governed by Web Access Control (WebID/ACL). Because pods are independently hosted, an offline pod yields partial rather than total failure, but full results require all pods to be reachable.

**Data interoperability:** In this architecture, each dataset is assigned a globally unique Uniform Resource Identifier (URI) and described using RDF aligned with shared ontologies like LHO. For instance, a specific cattle sample might be identified as *<http://www.purl.org/decide/LiveStockHealthOnto/LHO#Lab1CattleSample_0>.* Such URIs enable consistent interpretation and integration across distributed datasets, supporting semantic interoperability.

**Data reusability:** The federated access supports data reusability. Since each pod exposes machine-readable, semantically structured data using global identifiers, the same data can be queried for multiple purposes without duplication. Reuse is further enhanced by ontology-based annotation and metadata, which provide context and meaning to each data point
